# A retrospective cohort study comparing high and low balloon inflation pressure on technical success and patency for treating central venous lesions in patients on chronic hemodialysis

**DOI:** 10.1080/0886022X.2021.1975741

**Published:** 2021-09-09

**Authors:** Long Cui, Dan Gao, Xiaohan Lu, Zhao Gao, Hai Yuan, Fengqi Hu

**Affiliations:** Department of Nephrology, Xiangyang Central Hospital, Affiliated Hospital of Hubei University of Arts and Science, Xiangyang, Hubei, China

**Keywords:** High-pressure balloon, central venous stenosis, central venous occlusion, hemodialysis, percutaneous transluminal angioplasty

## Abstract

**Background:**

We aimed to analyze the success rates and the access patency rates at 12 months between patients on chronic hemodialysis with symptomatic central venous stenosis (CVS) or occlusion (CVO), receiving high or low balloon inflation pressure for treatment.

**Methods:**

We performed a retrospective study in which angioplasty balloons were inflated using a low-pressure or a high-pressure for the management of hemodialysis patients with CVS/CVO. The outcomes of this study were the success rate and the access patency rates at 12 months after balloon angioplasty, and the differences between groups were compared.

**Results:**

We included a total of 74 patients on hemodialysis and assigned them to the low-pressure or the high-pressure groups. Success rates in patients of the high-pressure group (94.12%) were higher than those in patients of the low-pressure group (67.50%) (*p* = 0.005). With a total of 59 patients with technical success, at 6 and 12 months after angioplasty, the rates of access patency in the low-pressure group were 68 and 48%, respectively; on the other hand, the primary patency rates in the high-pressure group were 86.67% (6-months) and 76.67% (12-months). The 6 and 12 months post-interventional patency rates were higher in patients of the high-pressure group than those in patients of the low-pressure group (*p* = 0.10 at 6 months an*d p* = 0.03 at 12 months).

**Conclusions:**

Compared to balloon angioplasty using a low inflation pressure, hemodialysis patients with CVS/CVO receiving angioplasty using a high inflation pressure have significantly higher technical success and 12-month patency rates.

## Introduction

The burden of end-stage renal disease requiring hemodialysis increases successively and vascular accesses are required for the continuation of hemodialysis. In these patients, access complication management is very important since the frequency of complications is high [1]. Dysfunctional hemodialysis vascular access is an important source of disease burden among patients on maintenance hemodialysis [1]. Among the spectrum of vascular access dysfunction, central venous stenosis (CVS) and central venous occlusion (CVO) are integral, since they prominently compromise the long-term survival of arteriovenous access. Both CVS and CVO have been found to occur in 5–50% of patients receiving chronic hemodialysis and constitute a difficult-to-treat central venous catheter (CVC)-related complication [2]. CVS is frequently asymptomatic, and the symptoms of CVS/CVO include local swelling involving affected extremities and the development of superior vena cava syndrome. These complications may compromise the efficiency of hemodialysis due to the loss of dialysis vascular access.

There are various types of treatment options available for CVS/CVO in patients on chronic hemodialysis. Guidelines from the Kidney Disease Outcomes: Quality Initiative (KDOQI) for vascular access recommended that asymptomatic CVS should not be treated [2]. Endovascular interventions, such as percutaneous transluminal angioplasty (PTA) and stenting (PTS) are the preferred treatments for symptomatic CVS/CVO. A recent meta-analysis revealed that, compared to PTS, PTA may increase primary patency rates after endovascular treatments of CVS/CVO in patients receiving chronic hemodialysis [3]. The KDOQI guidelines also suggest that PTA could be used as the first line of treatment for patients with symptomatic CVS/CVO [2]. In addition, prior studies demonstrated that the patency rates did not differ between patients with arteriovenous fistulae (AVF) or arteriovenous graft (AVG) stenosis receiving low-pressure or high-pressure balloon angioplasty [4,5]. However, to the best of our knowledge, whether high-pressure balloon PTA provided improved technical success and/or higher long-term patency rates compared with low-pressure balloon PTA in patients with CVS/CVO remains unclear. In this retrospective study of a single-center, we investigated and compared the technical success rates and the long-term patency rates of central veins in patients whose stenosis/occlusions were treated with low-pressure or high-pressure balloon dilation.

## Materials and methods

### Design of the current study

We retrospectively identified patients maintenance hemodialysis who developed CVS or CVO and were symptomatic, treated with non-compliant balloon angioplasty with low or high inflation pressure from January 2018 to January 2020. Those with symptomatic lesions, stenosis, or occlusions involving the superior vena cava, brachiocephalic veins, or subclavian veins were included. The diagnosis of CVS/CVO was made according to patients’ symptoms and diagnostic images, confirmed by angiographic films showing >50% diameter stenosis reduction relative to the diameters of the superior vena cava, brachiocephalic veins, or subclavian veins, which are the reference vessels [6]. Quantitative Vascular Analysis (QVA) was used to measure the vessel diameter. Those with intra-stent stenosis of their central veins or with a history of vasodilation for CVS/CVO were excluded.

In 2018, low-pressure angioplasty balloons (Bard Peripheral Vascular, Tempe, AZ, USA/Boston Scientific, Natick, MA, USA), with a rated burst pressure (RBP) 10–14 atm, were used in our institute for treating access stenosis. However, in May 2019, high-pressure balloons with an RPB 24–30 atm (Bard Peripheral Vascular) became available and were henceforth used routinely in the replacement of the original low-pressure catheters. The diameters of the high-pressure and low-pressure balloons used in this study are both 8–12 mm. Each patient was followed up every month after treatment, and their symptoms were monitored including edema of their arm, trunk, neck, and face, whether they had superior vena cava syndrome and their access function. A second PTA would be carried out once they had CVS/CVO again with symptoms later during the follow-up period. The current study complied with the Declaration of Helsinki and the protocol of our study was approved by the Ethical Committee of Xiangyang Central Hospital, Xiangyang, China (approval number 2018-011). Written informed consent was obtained from each patient.

### PTA procedure

A standardized angioplasty procedure was adopted for treating CVS/CVO. We used the low-pressure angioplasty balloons calibrated according to an RBP at 10–14 atm for those among the low-pressure balloon angioplasty group, while an RBP at 24–30 atm was adopted when we used the high-pressure angioplasty balloons in the high-pressure group. All angioplasty procedures were performed under local anesthesia, and we monitored the patients using an electrocardiogram and a pulse oximeter, and their blood pressure was measured. We performed sequential venograms to examine the locations and the severities of CVS/CVO. Any procedural complications including puncture site hematoma, venous perforation, and the development of hemothorax were recorded. The optimal balloon diameter was selected based on adding 0–1 mm to the measured diameter of the unaffected segments within the target veins. We advanced the balloon over the wire, centering the balloon over the stenotic segments for dilation. A balloon with 4–8 cm length and a suitable diameter was inflated twice for 60–90 s using an inflator (BasixCOMPAK, Merit Medical Systems, South Jordan, UT, USA) until the indentation of the balloon disappeared or when we reached the maximum RBP specified by the manufacturers.

In patients having an AVF/AVG, the lesion sites were accessed through appropriate puncture sites overlying their AVF/AVG under ultrasound guidance. A 6–8 F short vascular sheath (Terumo, Tokyo, Japan) was placed and an angiographic catheter was used to probe the stenosis**/**occlusion of the patients’ central veins with a 0.035-inch hydrophilic guidewire (Terumo). We then placed the guidewire and performed PTA. A double puncture technique (a femoral puncture in addition to a fistula puncture) was used if the lesion could not be managed easily. Femoral vein catheterization was performed under ultrasound guidance. A 0.035-inch hydrophilic stiff guidewire (Terumo) was used to cross the obstructive lesion, followed by the procedures outlined above. In patients with a tunneled and cuffed CVC, we performed venography through their CVCs. We also obtained sequential venograms from their central veins to the hemodialysis CVCs through peripheral veins. If the obstruction was located at sites neighboring the catheter, the catheter was removed assisted by a guidewire capable of dilating stenosis after inserting a 10 F jugular introducer. The hemodialysis catheter was then relocated through the dilated vein with the tip reaching the right atrium. We employed the double puncture technique if needed, as described above.

### Outcome definitions

Patency was defined if a central vein remained free of recurrent symptomatic CVS/CVO or free of any requirement for interventions to maintain central vein patency. Technical success was defined according to the presence of <30% residual stenosis after endovascular intervention and the resolution of all collateral vessels. To assess residual stenosis, the diameters of target lesions were measured digitally and divided by the reference vessels on a post-interventional angiogram. Bare metal stent or contralateral AVF/AVG/CVC were used when patients without reach technical success.

### Statistical analysis

The distributions of continuous variables were examined using a Shapiro-Wilk test. Data was described in means with standard deviation or medians (25–75% interquartile range). Continuous variables between groups were compared using a *t*-test or a Mann–Whitney *U* test. Categorical variables between groups were compared using a chi-square test. To facilitate subsequent analyses, each PTA procedure was treated separately. Twelve-month patency rates were compared between the high- and low-pressure groups using Kaplan–Meier techniques and log-rank tests. SPSS version 25.0 (IBM, Armonk, NY, USA) was used during all analyses. A *p*-value <0.05 was considered statistically significant.

## Results

### Baseline data of study participants

A total of 74 patients on chronic hemodialysis received PTA for their CVS or CVO. The clinical features of participants are provided in Table 1. In 53 AVF/AVG-using patients, the indications for PTA of CVS/CVO included an obstructed upper extremity venous outflow complicated by functional impairment, the development of superior vena cava syndrome, the emergence of indirect signs of insufficient hemodialysis doses (e.g., low *Kt*/*V*, *Kt*/*V* = the ratio of the urea clearance × time product to total body water), and/or edema involving their arm, trunk, neck, and face, malfunctioning or bleeding AVF/AVG. In 21 patients on hemodialysis through a tunneled cuffed CVC, the indications for using PTA to treat CVS/CVO included an obvious CVS/CVO-related CVC malfunction with or without symptomatic venous obstruction, presenting as regional swelling and/or unilateral superficial collateral veins.

**Table 1.** Baseline characteristics of patients within each group.

Characteristic	Total number, *n* (%)	High-pressure balloon angioplasty group	Low-pressure balloon angioplasty group	*p*-Value
Patients	74	34	40	/
Age (years)	/	65.50 (56.25, 75.75)	60.00 (54.00, 74.00)	0.31
Male sex, *n* (%)	28 (37.84%)	12 (35.29%)	16 (40.00%)	0.68
Diabetes mellitus, *n* (%)	16 (21.62%)	5 (14.71%)	11 (27.50%)	0.18
Vascular access time from creation to intervention (months)	/	24.00 (13.50, 48.00)	33.00 (7.00, 56.50)	0.85
Previous CVC, *n* (%)	53 (71.62%)	27 (79.41%)	26 (65.00%)	0.17
Vascular access, *n* (%)				
AVF/AVG	53 (71.62%)	22 (64.71%)	31 (77.50%)	
Tunneled, cuffed (long-term) CVC	21 (28.38%)	12 (35.29%)	9 (22.50%)	
Site of the stenosis/occlusion, *n* (%)				0.32
Right	26 (35.14%)	14 (41.18%)	12 (30.00%)	
Left	48 (64.86%)	20 (58.82%)	28 (70.00%)	
Location, *n* (%)				0.50
Superior vena cava	37 (50.00%)	19 (55.88%)	18 (45.00%)	
Brachiocephalic veins,	22 (29.73%)	10 (29.41%)	12 (30.00%)	
Subclavian veins	15 (20.27%)	5 (14.71%)	10 (25.00%)	
Nature, *n* (%)				0.92
Stenosis	57 (77.03%)	26 (76.47%)	31 (77.50%)	
Occlusion	17 (22.97%)	8 (23.53%)	9 (22.50%)	
Procedural success, *n* (%)	59 (79.73%)	32 (94.12%)	27 (67.50%)	0.005

AVF: arteriovenous fistulae; AVG: arteriovenous graft; CVC: central venous catheter.

**Figure 1.**
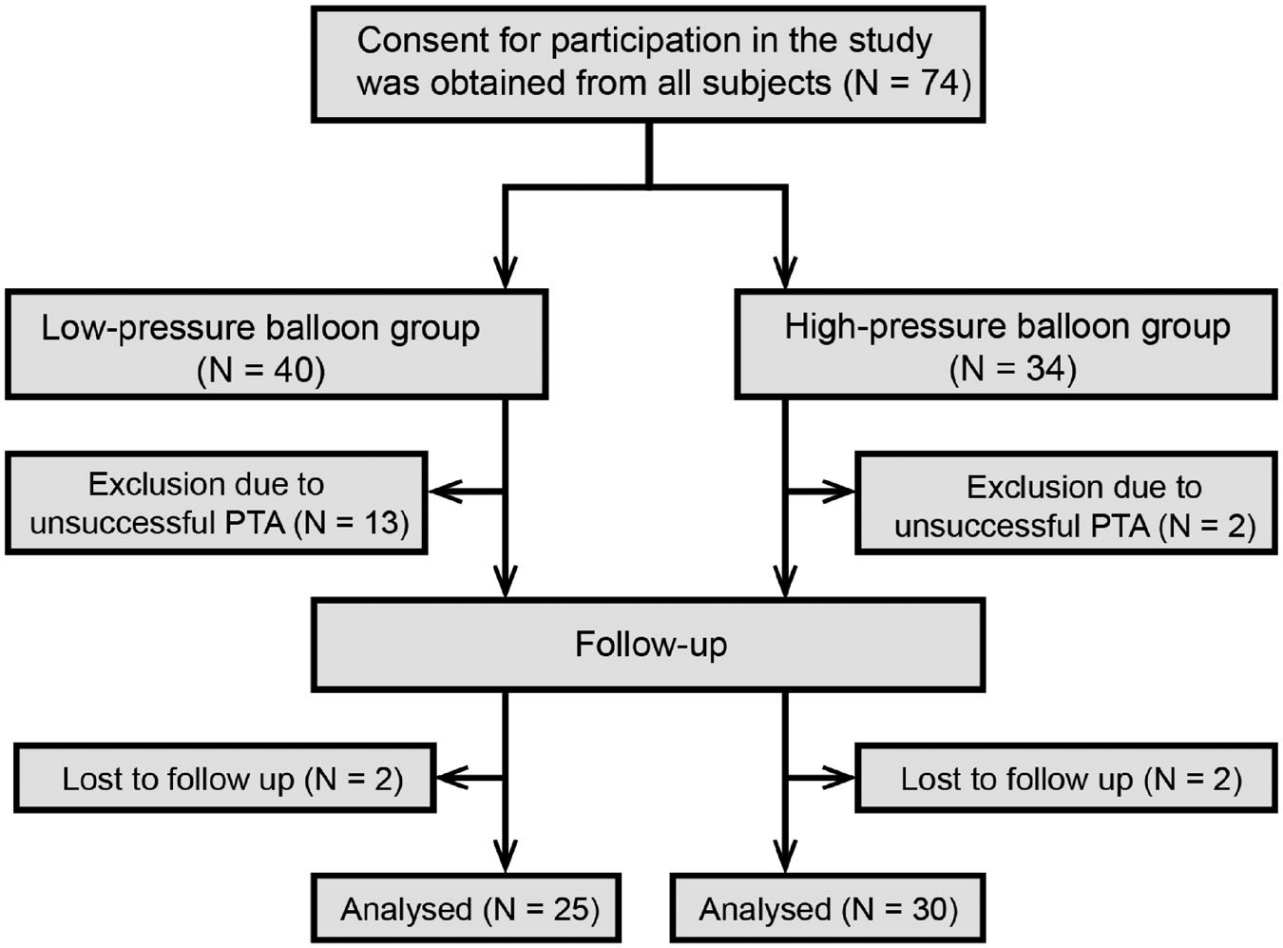
Flow chart for patient identification.

There was no significant difference between the low-pressure and high-pressure balloon angioplasty groups regarding age (*p* = 0.31), sex (*p* = 0.68), the prevalence of diabetes mellitus (*p* = 0.18), the duration between vascular access creation and intervention (*p* = 0.85), the types of hemodialysis vascular access (*p* = 0.22), the sites of stenosis**/**occlusion (left *vs.* right) (*p* = 0.32), the location of the stenosis**/**occlusion (*p* = 0.50) and the ratio of stenosis**/**occlusion (*p* = 0.92) (Table 1). The stenotic/occluded venous segments were mostly located on the left side (64.86%), with the majority affecting the superior vena cava (50.00%), brachiocephalic veins (29.73%), and subclavian veins (20.27%). Fifty-three patients had an AVF/AVG, while 21 had a tunneled cuffed CVC (Table 1). Thirteen and two patients in the low- and high-pressure arms, respectively, had an unsuccessful PTA procedure (>30% residual stenosis or failure to pass the obstruction site). Ultimately, 59 patients received a successful PTA, with a technical success rate of 67.50 and 94.12% in the low- and high-pressure arms, respectively. Significant differences were observed between the two groups regarding the procedural success rates (*p* = 0.005) (Table 1). Two patients in each group were not followed up at 12 months as a result of restenosis (Figure 1).

### Comparing the patency rates with technical success between the low- and high-pressure groups

**Figure 2.**
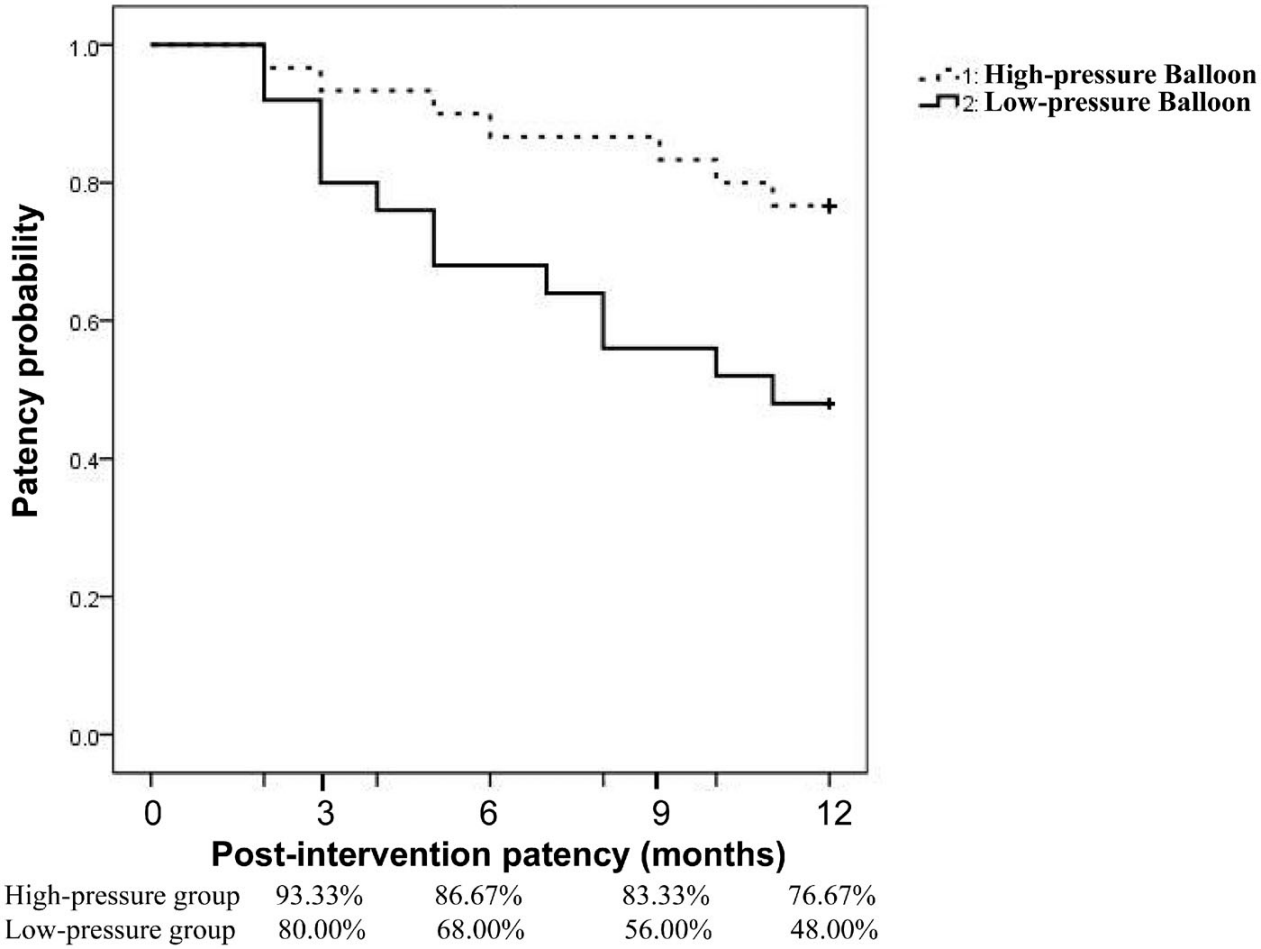
Kaplan–Meier curves of primary PTA patency rates between the high- and low-pressure balloon angioplasty arms. Patients who received successful PTA were included for calculating long-term patency rates. Each PTA procedure was treated separately. Comparisons between the low-pressure and high-pressure groups based on results from the Kaplan–Meier analysis and the log-rank test. The patency rate for using high-pressure angioplasty was higher than that of low-pressure balloons (*p* = 0.02).

The post-interventional patency rates of central veins in the low-pressure group with technical success were 80.00, 68.00, 56.00, and 48.00% at 3, 6, 9, and 12 months, respectively, while in the high-pressure group with technical success, patency rates were 93.33, 86.67, 83.33, and 76.67%, respectively. After comparison, we found that the 9- and 12-month patency rates in the low-pressure group were significantly lower compared to those in the high-pressure group (*p* = 0.03 and 0.03 for the 9- and 12-month results, respectively (Table 2). The detailed patency data is provided in Table 2. The trend of post-interventional patency among the groups is illustrated using the Kaplan–Meier technique (Figure 2) and compared using the log-rank test. Patients of the high-pressure arm had a higher patency rate at 12-months than those in the low-pressure arm (*p* = 0.02). The 12-month post-interventional patency rates were still significantly higher in patients of the high-pressure group after we excluded cases with subclavian vein involvement in both groups (Table 3).

**Table 2.** Patency rates at follow-up in two groups with technical success.

Time after PTA	Low-pressure		High-pressure		*p*-Value
	Patency (%)	95% CI	Patency (%)	95% CI	
3 months	80.00 (20/25)	0.64–0.96	93.33 (28/30)	0.84–1.00	0.14
6 months	68.00 (17/25)	0.49–0.87	86.67 (26/30)	0.74–1.00	0.10
9 months	56.00 (14/25)	0.36–0.76	83.33 (25/30)	0.69–0.97	0.03
12 months	48.00 (12/25)	0.28–0.68	76.67 (23/30)	0.61–0.93	0.03

CI: confidence interval; PTA: percutaneous transluminal angioplasty.

**Table 3.** Patency rates at follow-up in two groups with technical success after excluded cases with the subclavian vein.

Time after PTA	Low-pressure		High-pressure		*p*-Value
	Patency (%)	95% CI	Patency (%)	95% CI	
3 months	70.00 (14/20)	0.48–0.92	88.89 (24/27)	0.76–1.00	0.12
6 months	60.00 (12/20)	0.37–0.84	85.19 (23/27)	0.71–1.00	0.07
9 months	55.00 (11/20)	0.31–0.79	81.48 (22/27)	0.66–0.97	0.04
12 months	45.00 (9/20)	0.21–0.69	77.78 (21/27)	0.61–0.95	0.02

CI: confidence interval; PTA: percutaneous transluminal angioplasty.

### Complications

We did not observe any procedure-related complications including hematoma at the puncture site, venous perforation, or hemothorax, in the high- or low-pressure arms.

## Discussion

In this study, we aimed to analyze the effect of the balloon dilation pressure on the technical success and patency rates among patients on chronic hemodialysis with symptomatic CVS/CVO. We discovered that the technical success rates were 67.50 and 94.12% in the low- and high-pressure arms, respectively. We also found that the 12-month patency rates of patients within the high-pressure arm were higher than those of patients within the low-pressure arm. The results from our study indicate that high-pressure balloon angioplasty for CVS/CVO might improve technical success rates and post-interventional patency rates compared to low-pressure dilation among patients receiving chronic hemodialysis.

Hemodialysis vascular access dysfunction serves as an instrumental morbidity origin and has been shown to be responsible for 20–30% of the annual hospitalization in patients on chronic hemodialysis [7,8]. The main etiology of dysfunctional vascular access is known to be venous stenosis [9]. KDOQI guidelines for vascular access in patients receiving chronic hemodialysis recommend using balloon angioplasty as the mainstay of treatment for AVF/AVG stenosis and CVS/CVO [2]. Different balloons are available for the purpose of angioplasty, including the low-pressure balloon, the high-pressure balloon, the paclitaxel-coated balloon, and the cutting balloon. Some studies have shown that PTA using high-pressure balloons could improve technical success rates for correcting AVF/AVG stenosis compared to low-pressure ones [4,5]. On the other hand, other studies have reported no significant difference between PTA using low-pressure and high-pressure dilation for AVF/AVG stenosis regarding patency rates [4,5]. Our study further indicates the efficacy of high-pressure balloon angioplasty by showing an improvement of both technical success rates and post-interventional patency rates by high-pressure dilation compared with low-pressure for CVS/CVO.

The differences in patency rates may result from the discrepancy between the pathogenesis of AVF/AVG stenosis and that of CVS/CVO. The pathophysiology of AVF/AVG stenosis predominantly involves the formation of neointimal hyperplasia related to hemodynamic stimuli (in the case of AVF and AVG stenosis) and macrophage accumulation-triggered inflammation in response to foreign materials (in the case of AVG stenosis) [10]. CVS/CVO is frequently associated with the insertion of CVC for performing hemodialysis. The risk of developing CVS/CVO has been shown to increase in parallel to the number of previously inserted CVCs and the length of time during which CVCs are kept *in situ* [6]. In this study, we also found that 71.6% of patients with CVS/CVO had previously received at least one CVC placement (including temporary CVCs without tunneled cuffs and tunneled cuffed CVCs) and 28.4% had previously received a tunneled cuffed CVC placement. The site of CVS/CVO is related to the location of CVC [11]. In this study, the majority of CVS/CVO was located in superior vena cava may be related to that most patients have previous CVC, and in our center patients with CVC were inserted into the internal jugular vein. The pathogenesis of CVS/CVO, especially that of CVS/CVO related to CVC placement, is more complex than that of AVF/AVG stenosis. Firstly, CVS related to CVC placement involves endothelial damage and coagulation cascade activation [12,13]. Although tunneled cuffed CVC for hemodialysis has been implicated as a key player in the pathogenesis of CVS, non-tunneled CVC may similarly participate in CVS/CVO pathogenesis [11]. Secondly, the bio-incompatibility of CVC can induce inflammatory venous injuries [12,13]. Vascular endothelial injuries resulting from local inflammation involving central veins can lead to fibrosis development [2]. Thirdly, CVS/CVO can sometimes be idiopathic. Events of CVS/CVO without a prior history of CVC insertion have been associated with the presence of proximal vascular access with a high blood flow. Neointimal hyperplasia and CVS may emerge secondary to shear stress alterations and turbulence caused by the high blood flow inherent to AVF/AVG [13,14]. Finally, anatomical factors also play a role in the pathophysiology of CVS/CVO. Central veins are anatomically left-sided and can be inclined to develop stenosis/occlusion due to their longer and more tortuous course [11]. Prior studies disclosed a higher incidence of CVS/CVO after placing a left-sided CVC compared to a right-sided one [6]. We also found most CVS/CVO events involved left-sided central veins (64.9%). Since heterogeneity in lesions of CVS/CVO exists, especially with regard to the site of external bony compression overlying the subclavian vein, whose feasibility for treatment creates concern. Some investigators suggested that the trimming of the first rib or clavicular bone surgery might facilitate a better treatment efficacy before PTA compared to PTA alone for subclavian vein [12,15]. However, in the present study, the 12-month post-interventional patency rates were still significantly higher in patients of the high-pressure group after lesions of the subclavian vein were excluded in both groups.

According to the manufacturer’s instructions, the low-pressure balloon is composed of nylon, and the high-pressure balloon is composed of fiber-containing composite materials. Prior reports showed that PTA using a high-pressure balloon (RBP > 20 atm) could mechanically disrupt dense fibrotic tissue at the stenotic segments and improve the success rates of PTA [16]. Their findings could lend support to the observed higher technical success rates in the high-pressure dilation group for CVS/CVO in this study. Compared with primary stenotic lesions, the restenotic after PTA lesions are related to increase in fibroplastic proliferation and aggressive growth of endothelial cells within the venous [17,18]. The destruction of fibrotic tissue by the high-pressure balloon may delay the fibroplastic proliferation of venous, which may explain the reason for the reduction of symptomatic restenosis after the high-pressure balloon PTA. This result also explains the paclitaxel-coated balloons angioplasty with restenositic CVS seems to improve the patency rate compared to standard balloon angioplasty, in which paclitaxel have the effects of potent cytotoxicity, thereby show antiproliferative and inhibit the growth of endothelial cells [17–19].

The present study has its limitations. Firstly, it was retrospective in nature. Secondly, the study was done using data from a single-center and we had a modest number of patients. Thirdly, lesions of CVS/CVO exist heterogeneity. Finally, the mechanisms responsible for the observed higher post-interventional patency rates in the high-pressure group in this study remained inadequately elucidated.

In summary, we found that PTA using either a high-pressure balloon (RBP 24–30 atm) or a low-pressure balloon (RBP 10–14 atm) were both safe. Using high-pressure balloons could improve the technical success rates and post-interventional patency rates for CVS/CVO compared with procedures using low-pressure balloons. Given the better outcomes related to high-pressure balloons, we conclude that high-pressure angioplasty balloons can be recommended for the treatment of CVS/CVO among patients on chronic hemodialysis.
